# Barrel Jellyfish (*Rhizostoma pulmo*) as Source of Antioxidant Peptides

**DOI:** 10.3390/md17020134

**Published:** 2019-02-23

**Authors:** Stefania De Domenico, Gianluca De Rinaldis, Mélanie Paulmery, Stefano Piraino, Antonella Leone

**Affiliations:** 1 Istituto di Scienze delle Produzioni Alimentari, Consiglio Nazionale delle Ricerche (CNR-ISPA) Unit of Lecce, Via Monteroni, 73100 Lecce, Italy; stefania.dedomenico@ispa.cnr.it (S.D.D.); gianluca.derinaldis@ispa.cnr.it (G.D.R.); 2 Dipartimento di Biotecnologia, Chimica e Farmacia (DBCF), Università Degli Studi Di Siena, Via A. Moro, 2, 53100 Siena, Italy; 3 Département des Sciences et Technologies, Université de Lille, Cité Scientifique, F-59655 Villeneuve d’Ascq, France; melanie.paulmery@gmail.com; 4 Dipartimento di Scienze e Tecnologie Biologiche ed Ambientali (DiSTeBA), University of Salento, 73100 Lecce, Italy; stefano.piraino@unisalento.it; 5 Consorzio Nazionale Interuniversitario per le Scienze del Mare (CoNISMa), Local Unit of Lecce, Via Monteroni, 73100 Lecce, Italy

**Keywords:** invertebrate proteins, biological activity, antioxidants, collagen, pepsin hydrolysis, collagenase hydrolysis, oxidative stress, keratinocytes, cytotoxicity

## Abstract

The jellyfish *Rhizostoma pulmo*, Macrì 1778 (Cnidaria, Rhizostomae) undergoes recurrent outbreaks in the Mediterranean coastal waters, with large biomass populations representing a nuisance or damage for marine and maritime activities. A preliminary overview of the antioxidant activity (AA) of *R. pulmo* proteinaceous compounds is provided here based on the extraction and characterization of both soluble and insoluble membrane-fractioned proteins, the latter digested by sequential enzymatic hydrolyses with pepsin and collagenases. All jellyfish proteins showed significant AA, with low molecular weight (MW) proteins correlated with greater antioxidant activity. In particular, collagenase-hydrolysed collagen resulted in peptides with MW lower than 3 kDa, ranging 3–10 kDa or 10–30 kDa, with AA inversely proportional to MW. No cytotoxic effect was detected on cultured human keratinocytes (HEKa) in a range of protein concentration 0.05–20 μg/mL for all tested protein fractions except for soluble proteins higher than 30 kDa, likely containing the jellyfish venom compounds. Furthermore, hydrolyzed jellyfish collagen peptides showed a significantly higher AA and provided a greater protective effect against oxidative stress in HEKa than the hydrolyzed collagen peptides from vertebrates. Due to a high reproductive potential, jellyfish may represent a potential socioeconomic opportunity as a source of natural bioactive compounds, with far-reaching beneficial implications. Eventually, improvements in processing technology will promote the use of untapped marine biomasses in nutraceutical, cosmeceutical, and pharmaceutical fields, turning marine management problems into a more positive perspective.

## 1. Introduction

In European populations, jellyfish evoke unpleasant or disgusting feelings, meanwhile in Asia, they are recognized as an important source of bioactive compounds used in traditional food and medicine [1].

Variations in water mass, high salinity, and warm temperature associated with the current global climatic change, in combination with multiple anthropogenic impacts such as overfishing and coastal sprawl, led to increases in jellyfish peak abundances (blooms) and frequencies in the world’s oceans [2,3]. Jellyfish blooms usually negatively impact human health and activities in coastal waters [4,5,6]. Instead, marine gelatinous organisms should be regarded through a more positive perspective as new important bio-resource [7,8].

Known for its nutritional and medical value in the Chinese pharmacopeia, increasing attention has been pointed to medusozoan jellyfish as an untapped source of essential nutrients [9,10,11], novel bioactive metabolites, and lead compounds, so to have been recently appointed as novel food in western Countries [12,13,14,15].

In the last decade, different kinds of extracts obtained from several specimens of jellyfish were analyzed and many pharmacological activities were found: for instance, several studies have been focused on box jellyfish venoms, which contain a great variety of bioactive proteins and are shown to have hemolytic, cytotoxic, cardiovascular [16,17,18,19,20], neurotoxic [17,21,22,23], and anti-tumoral [24] activities, both *in vivo* and *in vitro*. Furthermore, similar analysis has been performed using extracts carried out on the whole jellyfish biomass. Jellyfish tissue components showed others biological activities linked to proteins components on both cell cultures and *in vivo*, such as anti-fatigue activity [25] cytotoxicity on cancer cells [10,26], apoptosis and anti-cancer effects [27], and antioxidant properties [11,28,29,30,31], as well as anti-microbial activity [32].

Studies on the biochemical composition of wild jellyfish biomass, however, have been available only in recent decades. Despite the large biomasses, the dry weight of most rhizostomeae jellyfish (Cnidaria, Scyphozoa) ranges from 2–5%, mainly composed of proteins, while carbohydrates and lipids represent minor components [11,33,34].

New biological functions are increasingly attributed to protein hydrolysates and derived peptides, obtained from vegetable and animal sources [35,36], including antihypertensive, antitumoral, antiproliferative, hypocholesterolemic, anti-inflammatory and antioxidant activities [37,38]. Bioactive peptides are released during food processing or as result of enzymatic or chemical hydrolyses. Their functions are largely influenced by the nature of proteins, hydrolytic enzymes, enzyme-substrate ratio, temperature and time of reaction. All these conditions affect the molecular weight and the amino acid composition of peptides and, as a consequence, their activities [39].

Scientific evidence has clearly indicated a link between oxidative stress and various chronic diseases and aging, involving both intrinsic and extrinsic sources of Reactive Oxygen Species (ROS) as key mechanisms of these processes [40,41,42].

Recently, attention has been paid to antioxidant activity associated to a single or mixtures of molecules deriving from natural sources, according to their recognized role in the prevention of oxidative stresses mechanisms associated with numerous degenerative diseases, such as diabetes, cardiovascular and neurodegenerative disorders, and cancer [43,44,45,46]. Natural antioxidants may exhibit a reduced potential health hazard compared with synthetic compounds and they are already used in food industry as dietary supplements and in pharmaceuticals or cosmeceutical products, as replacement for synthetic antioxidants. Indeed, protein hydrolysates from plant and animal sources have been found to possess strong antioxidant activities [47,48,49,50].

Enriched by an enormous but still poorly explored biodiversity, the oceans represent an immense reservoir of bioactive peptides [51], extracted from a variety of diverse marine organisms, from invertebrates such as sponges, tunicates, bivalves, cephalopods [51,52,53,54,55] to vertebrates, such as the hairtail fish *Trichiurus lepturus* [56]. Protein hydrolysates of seafood and their by-products are known to have different functional properties and great potential for nutraceutical and pharmaceutical applications [57] including oxidative stress protection. Moreover, a considerable number of these marine peptides have been identified and characterized: they are generally short with low molecular weight [58]; they seem resistant to gastrointestinal hydrolysis, enhancing their absorption in intact form [56,58,59,60].

Among marine invertebrates, jellyfish could represent an abundant source of new bioactive peptides, due to the high protein content, especially in collagen that accounts for up to 40–60% of dry weight [34,61]. Collagen is a group of fibrous proteins and it is the main component of extracellular matrix with a structure highly conserved and characterized by triple helical structure with repeating sequence of Gly-X-Y, where generally X is proline and Y is hydroxyproline [62,63]. Jellyfish collagen shares several features with its vertebrate counterpart that makes it highly biocompatible [64]. A few recent research studies are focused on jellyfish collagen and hydrolyzed collagen, which was shown to have biological activities as angiotensin-converting enzyme inhibitory action [65], immune-stimulation effects [66], anti-fatigue [25] and antioxidant proprieties [25,65]. Furthermore, collagen molecules extracted from the Mediterranean Sea barrel jellyfish, *Rhizostoma pulmo* [67], seem to have an effect on human cell comparable to the mammalian type I collagen [61].

In this study, novel information is provided about proteins extraction and hydrolyzed peptides isolation from *Rhizostoma pulmo*, one of the most abundant jellyfish species along the Mediterranean coasts. This jellyfish is characterized by a rather harmless envenomation potential for humans, and by typically possessing a greater body size and body texture than other scyphozoan jellyfish (e.g., compared with the highly watery moon jellyfish, *Aurelia*). Several molecular weight proteins fractions, including hydrolyzed collagen peptides, were here analyzed for their antioxidant activity *in vitro,* including on human keratinocytes cultures under oxidative stress conditions. Our results strongly suggest that the Mediterranean Sea barrel jellyfish, due to its metagenetic life cycle and high proliferative potential, may well represent a sustainable source for natural antioxidant bioprospecting and, more generally, for the isolation of bioactive compounds.

## 2. Results and Discussion

### 2.1. Proteins Content and SDS-PAGE Separation

The lyophilized *Rhizostoma pulmo* whole jellyfish samples (umbrella and oral arms) were subjected to aqueous protein extraction by phosphate-buffered saline (PBS) to separate the hydro-soluble fractions from the insoluble ones, which were eventually exposed to a two-step sequential enzymatic digestion.

Soluble proteins and hydrolysed peptides were molecular weight (MW)-fractionated by membrane filtration and each fraction was analysed for antioxidant activity and for their effect on cultures of human keratinocyte adult (HEKa) cells. Membrane ultrafiltration was here used as the first step in the *R. pulmo* peptide purification, as reported for purification of bioactive peptides from the edible jellyfish *Rhopilema esculentum* [68] and other invertebrates [58].

The average concentration of *R. pulmo* proteins was 34.1±2 mg/g of dry weight (DW) (Table 1), with a small majority (56%) composed by of PBS-soluble peptides and the remaining fraction (44%) by insoluble proteins. In order to roughly characterize the protein fractions, hydrosoluble proteins (SP) were separated in four sub-fractions at different molecular weight (MW) ranges: higher than 30 kDa (SP > 30), between 30 and 10 kDa (SP 10–30), between 10 and 3 kDa (SP 3–10), and lower than 3 kDa (SP < 3). Near 93% of soluble proteins had MW higher than 30 kDa, whereas only 0.5% of the total SP showed MW between 3 and 10 kDa; approximately 4% of the total SP had MW between 10 or 30 kDa and less than 3% with MW < 3 kDa. The latter sub-fraction was no further considered because of the high salt content. In our samples, the largest sub-fraction—i.e., containing peptides with high MW—most likely includes proteinaceous components of the jellyfish mucus, which was found to have MW higher than 40 kDa in various jellyfish species and other coelenterates [69,70,71]. In agreement with this hypothesis, the mucus fraction of the jellyfish *Aurelia coerulea* contained proteins falling within three MW ranges, i.e., 100–250 kDa, 50–100 kDa and 37–50 kDa, while the tissue proteins were dispersed in a wider range [71]. Interestingly, hydroalcoholic extracts of the zooxanthellate jellyfish *Cotylorhiza tuberculata* showed only low MW proteins ranging 10–14 kDa [10].

**Figure 1:**
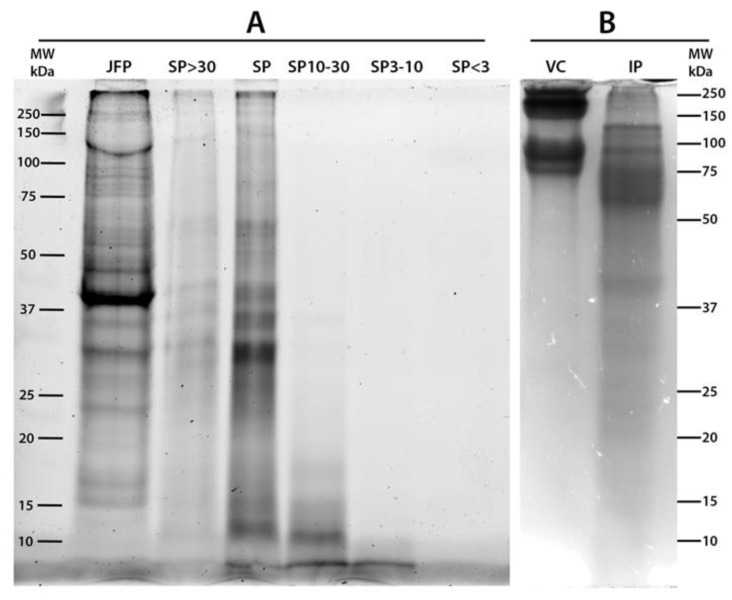
SDS-PAGE analysis of *Rhizostoma pulmo* jellyfish soluble proteins (20 µg) imaged with ChemiDoc MP Imaging System (**A**) and stained with Comassie Brillant Blu (**B**). (**A**) JFP, Total Jellyfish Proteins; SP > 30, Soluble Protein fraction with MW > 30 kDa; SP, Total SP; SP 10–30, Soluble Protein fraction 10 < MW < 30 kDa; SP 3–10, Soluble Protein fraction with 3 < MW < 10 kDa; SP < 3, Soluble Protein fraction with MW < 3 kDa; (**B**) VC, Calf skin Collagen (VC); IP, Insoluble Proteins; MW = Molecular-weight size marker.

The total jellyfish proteins (JFP), total soluble proteins (SP), SP sub-fractions with MW > 30 kDa (SP > 30), between 10 and 30 kDa (SP 10–30), between 3 and 10 kDa (SP 3–10) and <3 kDa (SP < 3) were analyzed by SDS-PAGE (Figure 1). A commercial purified vertebrate collagen from calf skin (vertebrate collagen, VC) was also analyzed as standard collagen (line 7). To identify the protein fractions with high sensitivity, *R. pulmo* soluble protein fractions were separated by SDS-PAGE and visualized by stain-free system (Figure 1A) and by the standard Coomassie Brillian Blue (CBB) staining (Figure 1B). The 2,2,2-Trichloroethanol (TCE) gel and Stain-Free system [72,73] allowed a higher sensitivity as compared to standard CBB staining, except for proteins with low tryptophan content, such as collagen, since the staining is based on the reaction among the tryptophan residues and trihalo compounds in the gel (see Materials and Method). The calf skin collagen (type I collagen) was considered as control also for its similarity with the collagen isolated from the jellyfish *R. esculentum* [74]. Bovine collagen (VC) is clearly detected in Figure 1B by CBB staining method due to the specific mechanism of staining. The lyophilized whole jellyfish sample (JFP, lane 1 Figure 1A) shows a large number of polypeptides in a wide MW range, and two main bands: at apparent MW about 39 kDa and a large, unstained band of about 150–250 kDa. The latter could be related to largely insoluble collagen proteins. Both these proteins seem to be insoluble in aqueous solution as they are not present in the total SP (lane 3) and in SP > 30 (lane 2) fractions. Therefore, the total SP sub-fraction (lane 3) is composed by proteins in a wide range of apparent MW with five main bands at about 60 kDa, 40 kDa, 35 kDa, 30 kDa and 12 kDa. Except for the latter band (12 kDa), all bands are also present in the SP > 30 fractions. Similar protein diversity at wide range of MW was found in aqueous extracts from deep-sea jellyfish [26]. Although faintly visible, the electrophoretic separation of proteins with apparent MW ranging 10–30 kDa (SP 10–30), 3–10 kDa (SP 3–10) and <3 kDa (SP < 3) is shown by lanes 4, 5 and 6, respectively. In Figure 1B the total insoluble jellyfish proteins are also separated by SDS-PAGE showing proteins in a wide apparent MW range, mainly in MW > 50 kDa.

After aqueous extraction, the insoluble proteins (IP) were submitted to sequential digestions with pepsin followed by collagenase, as in Leone et al. [11]. Hydrolysed peptides were sub-fractionated by membrane filtration to obtain peptides at different MW ranging 10–30 kDa, 3–10 kDa and <3 kDa.

In order to obtain reproducible and relatively easy to purify compounds, in each step a single commercial protease was used for the enzymatic hydrolysis. Single-protease hydrolysis is simpler when compared with a combination of several proteases and likely allows a better control of the physico-chemical conditions of the digestion, providing a relatively controlled composition of the resulting peptides [75]. These were then separated by membrane filtration in sub-fractions at different molecular weight (10–30, 3–10 and <3 kDa). All sub-fractions, soluble proteins and not-hydrolysed components were assayed *in vitro* for their antioxidant activity (AA) as a term of reference of the possible content of active substances.

### 2.2. In Vitro Antioxidant Activity of Soluble and Hydrolysed Protein Fractions

Increasing scientific evidence demonstrates that peptides with antioxidant properties can be obtained from marine vertebrate and invertebrate proteins, hydrolysed proteins, seafood by-products [11,56,58,60,76] as well as from terrestrial animal by-products [77]. In the present study, a preliminary overview of the antioxidant capacity of the Mediterranean Sea barrel jellyfish *Rhizostoma pulmo* proteins is provided. The soluble jellyfish proteins, fractionated according to their MW, as well as the insoluble proteins, both native and enzymatically hydrolysed and subsequently fractionated by MW, were analysed for their antioxidant activity (AA). The radical scavenging activity was evaluated by the ABTS assay, widely used as a screening assay for natural antioxidant compounds [78,79].

**Figure 2:**
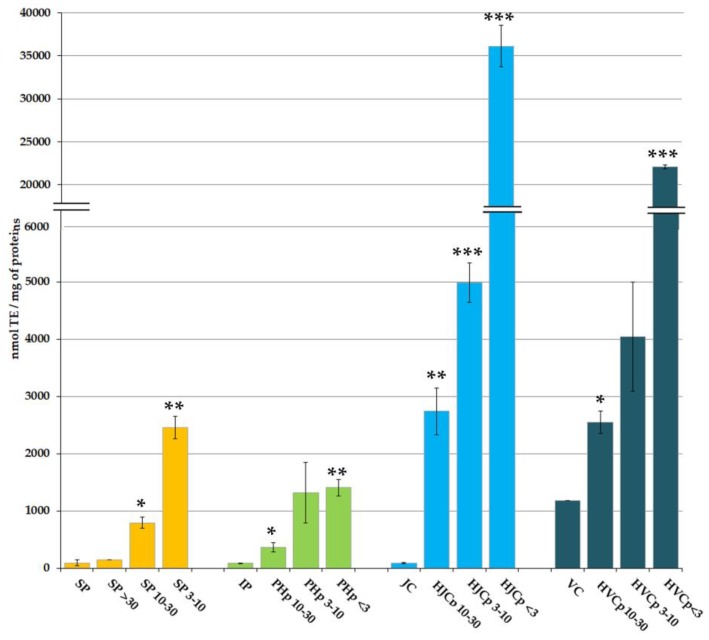
Antioxidant activity of *Rhizostoma pulmo* proteins. SP, Aqueous soluble proteins; IP, Insoluble proteins; JC, jellyfish collagen; VC, vertebrate (bovine) collagen. SP > 30, Soluble proteins with MW > 30 kDa; SP 10–30, Soluble proteins with MW 10–30 kDa; SP 3–10, Soluble proteins with MW 3–10 kDa; PHp 10–30, pepsin hydrolysed proteins with MW 10–30 kDa; PHp 3–10, pepsin hydrolysed proteins with MW 3–10 kDa; PHp < 3, pepsin hydrolysed proteins with MW < 3 kDa; HJCp 10–30, hydrolysed jellyfish collagen with MW 10–30 kDa; HJCp 3–10, hydrolysed jellyfish collagen with MW 3–10 kDa; HJCp < 3, hydrolysed jellyfish collagen with MW < 3 kDa; HVCp 10–30, hydrolysed vertebrate collagen with MW 10–30 kDa: HVCp 3–10, hydrolysed vertebrate collagen with MW between 3 and 10 kDa; HVCp < 3, hydrolysed vertebrate collagen with MW < 3 kDa. Data are the mean values of six independent experiments performed in three technical replicates. The antioxidant activity is expressed as nmol of TE per mg of protein ± standard deviation. Student’s t-test * *p* < 0.05, ** *p* < 0.01 and *** *p* < 0.001.

The AA of the considered protein fractions (Figure 2), expressed as nmol of Trolox equivalents (TE) per milligram of proteins, is remarkably higher in protein fractions with low MW than in fractions at high MW. The same AA pattern was observed both for PBS-soluble proteins and for the enzymatically hydrolysed peptides derived from insoluble proteins.

The AA evaluated in PBS soluble total protein (SP) was 92.9 nmol TE/mg of proteins, not significantly different from the AA measured in the fraction containing only high MW proteins (SP > 30) (145.0 nmol TE/mg of proteins). The peptides present in the fractions SP 10–30 and SP 3–10 showed AA values of 792.3 and 2459.0 nmol TE/mg of proteins, respectively. Both were significantly higher (*p* < 0.05 and *p* < 0.01, respectively) than AA values from SP and SP > 30 sub-fractions, and inversely proportional to the MW of peptides, demonstrating an enrichment in antioxidant compounds in low MW soluble protein sub-fractions.

Proteins insoluble in PBS (IP) showed an AA of 83.2 nmol TE/mg of proteins that was not significantly different from the extractable proteins; however, pepsin digestion produced a variable content of antioxidant peptides.

Depending on the source of protein and degree of hydrolysis, small MW sub-fractions of hydrolysed proteins were already demonstrated to possess the strongest AA activity [52,53,54,55,68,79,80], i.e., higher than high MW protein sub-fractions. Pepsin is able to hydrolyse a wide range of proteinaceous components including non-triple helical domains of collagen [81,82]. Therefore, pepsin digestion has been used here to aid the collagen solubilisation process [83,84,85]: indeed, the resulting extract consists mainly of non-collagen proteins and atelocollagen. In addition, pepsin cleaves peptides specifically in telopeptide region of collagen, which are non-helical ends; thus, by hydrolyzing some non-collagenous proteins, the pepsin treatment increases the purity of collagen and likely reduces its antigenicity [86]. In addition, the proteolytic effect of 2% pepsin (i.e., the concentration used here) cleaved cross-linked molecules without damaging the triple helix [87].

In our experiments, pepsin hydrolysed peptides (PHp) were separated by membrane filtration in sub-fractions containing peptides with different MW ranges: MW > 30 kDa, MW 10–30 kDa, MW 3–10 kDa and MW < 3 kDa. The fraction with peptides at highest MW (PHp > 30 kDa) was not further considered because it contained the pepsin enzyme (theoretical MW 34.5 kDa).

The AA values measured in the fractions PHp 10–30, PHp 3–10 and PHp < 3 were 364.9 nmol TE/mg, 1321.7 nmol TE/mg, and 1403,8 nmol TE/mg of proteins, respectively (Figure 2). Comparing the hydrolysed peptides with the parent proteins, AA values in PHp 10–30, PHp 3–10 or PHp < 3 resulted respectively four, fifteen, or sixteen times higher than the AA measured in the IP fraction.

Undigested insoluble proteins, after pepsin hydrolysis, likely consist of helical domains of collagen presumably inaccessible to pepsin action. As demonstrated in Leone et al. [11], bacterial collagenase was able to digest the insoluble protein fraction remained after pepsin hydrolysis, confirming its nature. Peptides derived from the *R. pulmo* proteins after collagenase digestion ranged MW 20–70 kDa, with two main bands at about 35 kDa and 50 kDa [11] In the present work, a fractionation of the collagenase-digested peptides was performed by membrane filtration, and four fractions were obtained at different MW range: MW > 30 kDa (HJCp > 30), MW 10–30 kDa (HJCp 10–30), MW 3–10 kDa (HJCp 3–10) and MW < 3 kDa (HJCp < 3). The commercial purified bovine collagen from calf skin (vertebrate collagen, VC) was also subjected to the same sequential pepsin-collagenase hydrolysis and MW-fractionation (HVCp > 30, HVCp 10–30, HVCp 3–10 and HVCp < 3) and was used as standard comparison with vertebrate collagen. The fractions HJCp > 30 and HVCp > 30 were not further considered because they contained the collagenase enzymes ranging in MW 68–130 kDa (as stated by the manufacturer). Proteins not hydrolysed by pepsin, mainly consisting of jellyfish collagen (JC) showed a very low AA (88 nmol TE/mg of proteins), which value was similar to that of undigested total protein (IP). The AA of not-hydrolysed bovine collagen (VC) was 1182 nmol TE/mg, but we could not determine whether it is an intrinsic characteristic or if the high AA value was due to the presence of other peptides.

The AA measured in the sub-fractions containing collagenase-hydrolysed jellyfish peptides resulted significantly higher as compared to proteins before collagenase digestion, namely 2741, 4980 and 36129 nmol TE/mg for proteins in the fractions HJCp 10–30, HJCp 3–10 and HJCp < 3, respectively. The AA of the fractions of peptides derived from vertebrate collagen were significantly lower as compared to jellyfish protein fractions, namely 2543, 4045, and 22,092 nmol TE/mg for peptide fractions HVCp 10–30, HVCp 3–10 and HVCp < 3, respectively. The main difference was between the fractions containing the smallest peptides, i.e., HJCp < 3 and HVCp < 3: AA appeared almost 2 times higher in low MW jellyfish-derived peptides than in low MW vertebrate-derived peptides. It is remarkable that, again, the hydrolysed peptides with lower MW showed higher AA in the order HJCp < 3 >>> HJCp 3–10 >> HJCp 10–30 >> JC. It is also notable that the value of AA of the smallest jellyfish peptides (HJCp < 3) was more than four hundred times the AA of collagenase-undigested peptides, the HJCp 3–10 more than twelve times higher and the HJCp 10–30 more than seven times higher. Similar differences, but less sharp, were evident in in vertebrate collagen fractions. These data confirmed and strengthened our previous findings about a higher AA of jellyfish collagens (from three species: *Aurelia coerulea*, *Cotylorhiza tuberculata* and *Rhizostoma pulmo*) as compared to chicken sternal cartilage collagen (Type II collagen) [11]. All jellyfish peptides derived from collagenase digestion showed considerably higher AA as compared to both peptides from pepsin digestion and PBS extracted (not hydrolysed) peptides [11].

The enzymatic hydrolysis of jellyfish proteins provided antioxidant peptides that can be further tested for their activity in cell culture systems. The enzymatic hydrolysis seems to be the most efficient method to produce homogeneous bioactive peptides, useful for further purification steps. As in this case, using specific enzymes and controlled reactions would help to release more homogeneous bioactive fragments than the chemical hydrolysis [88].

Although aqueous extraction can allow the solubilisation of antioxidant molecules other than proteins [11], the AA measured in the PBS extract and its fractions was lower as compared to hydrolysed fractions of the aqueous insoluble proteins. Therefore, the non-structural hydro-soluble proteins, the protein moiety of the mucus and maybe the proteinaceous toxins related to the abundant presence of nematocysts in the ectoderm and in the mucus of *R. pulmo* altogether play a marginal role in providing antioxidant activity compared to insoluble proteins. Although in this work the lyophilized whole jellyfish was considered, this finding seems to be in agreement with our recent finding that compounds from *R. pulmo* whole fresh jellyfish freely soluble in the PBS medium have low or no antioxidant activity [15].

Among *R. pulmo* hydrolysed proteins, the lower AA of pepsin-hydrolysed peptides, as compared to collagenase-digested proteins, could be due to the amino acid composition of the non-helical collagen as well as to other non-structural proteins of the jellyfish tissues. In addition, the pepsin-hydrolysed fractions could have a less homogeneous composition. Indeed, the activities of protein hydrolysates can be influenced by the amino acid composition, degree of hydrolysis, peptide size, peptide sequence, and type of used enzymes [65]. It is reasonable to assume that the differences in antioxidant activity could be related to differences in other biological activities.

### 2.3. Effect Jellyfish Proteins on HEKa Cell Cultures

#### 2.3.1. Effect of Soluble Protein Fractions on HEKa Cell Cultures

**Figure 3:**
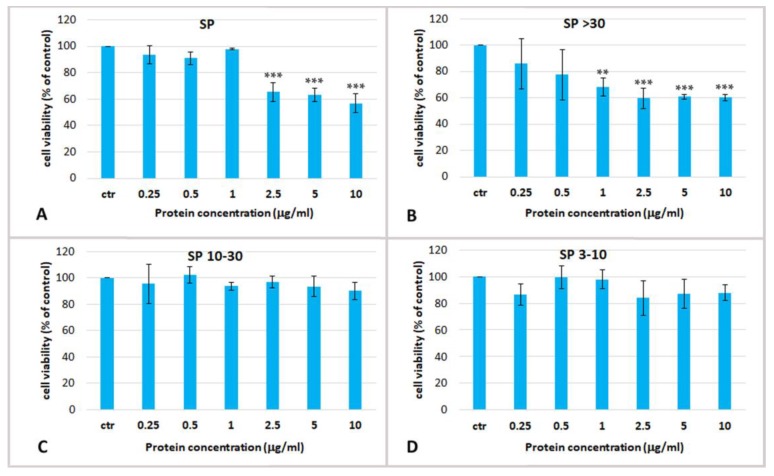
Cell viability of human epidermal keratinocytes (HEKa) treated for 24 h with different concentrations of (**A**) total PBS jellyfish extract (SP); (**B**) extract fraction containing molecules with a MW > 30 kDa (SP > 30), or (**C**) with MW 10–30 kDa (SP 10–30) or (**D**) MW 3–10 kDa (SP 3–10). Data are mean values of six independent experiments performed in five technical replicates, ±standard deviation. Ctr, control. ANOVA statistic test followed by Dunnett’s post-test was used to compare each treatment with the control, * *p* < 0.05, ** *p* < 0.01 and *** *p* < 0.001.

To determine the potential biological effect of aqueous soluble proteins of *R. pulmo*, dose-response experiments were carried out using total soluble extract and their derived fractions on cultured Human Epidermal Keratinocytes isolated from adult skin (HEKa). To verify and compare the possible cytotoxic effect HEKa cultures were treated with different jellyfish protein concentrations ranging from 0.25 to 20 µg/mL for 24 h, and the effect on cell viability was measured by MTS assay. The cell viability (Figure 3) was measured in HEKa cell cultures after treatment with the PBS whole extract (SP), and its fractions SP > 30, SP 10–30, SP 3–10. As already mentioned, the fraction containing compounds with MW lower than 3 kDa was not considered because of its high salt content, which could be cytotoxic *per se*. Figure 3 shows that only the whole jellyfish aqueous extract (SP) and its derived fraction containing proteins with MW > 30 kDa (SP > 30) were cytotoxic at the assayed concentrations. SP was able to reduce the cell viability to about 60% of the control at concentrations higher than 2.5 µg/mL, while SP > 30 exerted its cytotoxic effect from the concentration of 1 µg/mL. The fractions of jellyfish soluble proteins with MW ranging 10–30 kDa and 3–10 kDa were both non-cytotoxic even at the highest tested concentration (Figure 3C,D). The half maximal inhibitory concentration (IC_50_) for SP and SP > 30 fractions was determined at 2.7 ± 1.5 µg/mL and 1.01 ± 0.06 µg/mL, respectively. Therefore, cytotoxic soluble compounds seem to be proteins at MW > 30 kDa, as the fraction SP > 30 resulted enriched of toxic compounds.

Different fractions of a hydro alcoholic extract from *Cotylorhiza tuberculata* were found non- toxic for HEKa cells until a concentration of 80 µg/mL while they were cytotoxic for breast cancer cells MCF7 [10]; however, the nature of the extracted compounds could be very different due to the solvent and fractionation method used.

Various molecules can be responsible of the cytotoxicity detected here on HEKa cell cultures; indeed, the aqueous extract of *R. pulmo* contains soluble proteins including nematocyst venom. Nematocysts are subcellular organelles produced by highly specialized mechano-sensory nerve cells, the nematocytes [89]. Functional nematocytes are distributed in the ectodermal layer of cnidarian tissues, at high concentrations particularly over tentacles and oral structures. Upon mechanic or chemical signals, each nematocyte can fire a syringe-like filament injecting a mixture of proteinaceous and non-proteinaceous compounds produced and stored in the nematocysts [5]. Generally, the toxicological properties of jellyfish venoms are species-specific: *R. pulmo* it is commonly considered mild stinger to humans since its effect is no more than a burning sensation. However, the severity of envenomation depends on the number of discharged nematocytes and the affected body part. Interestingly, nematocysts are also extremely abundant in the mucus of *R. pulmo* (Leone, personal observation), possibly released as defensive mechanism following interspecific contacts.

Rhizolysin, a high molecular weight cytolysin of 260 kDa was isolated from the nematocysts of *R. pulmo* [90] and a 95 kDa metalloproteinase named rhizoprotease was identified in a tentacle extract fraction [91]. In *R. pulmo* a cytotoxic and hemolytic activity was also detected in tissue isolates free of nematocytes [92]. A number of venom proteins were characterized for their MW by SDS-PAGE separation: proteins with anticoagulant activity extracted from tentacles of *Aurelia* sp. (as *A. aurita*) showed MW ranging 50–160 kDa [93]; toxins with haemolytic activity with apparent MW of 42 kDa [94], 43 kDa and 45 kDa [95] were isolated from *Alatina* (*Carybdea*) *alata*; several nematocyst venom proteins with approximate MW of 35, 50, 55 and 100 kDa were found in *Chrysaora achlyos* [96]. FPLC gel filtration chromatography allowed the separation of venom proteins with molecular mass of about 102–107 kDa from the nematocysts of *Carybdea marsupialis* [97] and proteins peaks at 85 and 40 kDa obtained from the crude toxin of *Rhopilema nomadica* [98].

By functional assay (using fibrinolytic activity in zymography assay), Bae et al. [99] reported venom proteins of *Nemopilema nomurai* as characterized by MW of approximately 70, 35, 30, and 28 kDa. These authors compared *N. nomurai* with *Aurelia aurita* venoms, with similar banding patterns, distributed in 60–80 kDa and 25–37 kDa size bands, and with the siphonophoran *Physalia physalis* venom, with MW > 25 kDa.

**Figure 4:**
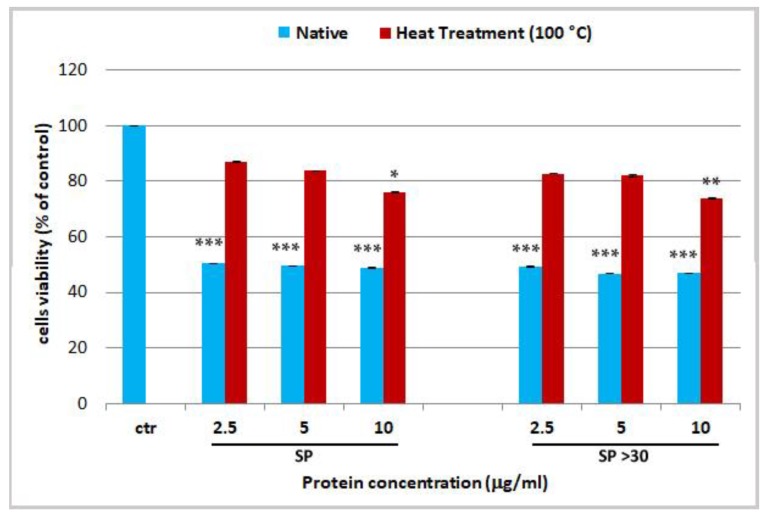
Cell viability of human epidermal keratinocytes (HEKa) treated with different concentrations of soluble proteins (SP) and soluble proteins with MW > 30 kDa (SP > 30), not treated (Native) and heat-denatured for 10 min at 100 °C (Heat Treatment 100 °C). Data are mean values of six independent experiment ± SD. Statistical analysis was carried out by ANOVA test followed by Dunnett’s post-test, * *p* < 0.05, ** *p* < 0.01 and *** *p* < 0.001.

In the present work, in order to verify the presence of proteinaceous venom in our extracts, the two cytotoxic fractions (SP and SP > 30) were heat-denatured by exposure to 100 °C for 10 min before their administration on cells. A loss of cytotoxicity on HEKa was observed after heat treatment of those fractions (Figure 4). This activity reduction was mitigated at the highest tested protein concentrations of 10 µg/mL in both, SP and SP > 30, with *p*-values of *p* < 0.05 and *p* < 0.01, respectively. The difference was likely due to the enrichment of toxins in the SP > 30 fraction, after membrane filtration, as compared to the whole extract (SP). In addition, as the cytotoxicity has not completely declined, at least at the highest concentration, some heat-resistant proteins should be still present. This confirms previous observations on the fibrinogenolytic activity of *R. pulmo* tentacle extract, which was significantly reduced but not abolished by heat treatment for 1 min at 100 °C [91], suggesting that some active components of the *R. pulmo* extracts are not completely thermolabile.

Our findings, together with literature data, suggest that a simple washing in aqueous solutions and the separation of high molecular weight proteins from the extract, e.g., by membrane filtration, could represent a crucial strategy for both removing possible toxic compounds from jellyfish extracts and to concentrate potentially bioactive soluble compounds. This basically simple procedure could be an easy starting point for the isolation of *R. pulmo* venom and for the development of a processing method of jellyfish biomasses suitable for isolation and characterization of potentially active soluble components, useful as nutraceutical and cosmeceutical ingredients.

#### 2.3.2. Effect of Hydrolysed Proteins on HEKa Cell Cultures

The fractions containing hydrolyzed jellyfish peptides were tested for their activity on HEKa cultures. The fractions containing jellyfish peptides with MW > 30 kDa, PHp > 30 and HJCp > 30 were not assayed because of the occurrence of the enzymes pepsin and collagenase, respectively.

**Figure 5:**
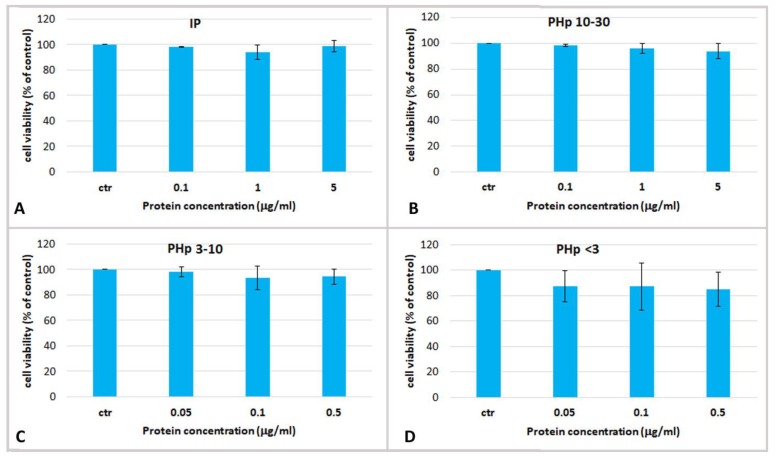
Cell viability of human epidermal keratinocytes (HEKa) treated with different concentrations of (**A**) insoluble jellyfish proteins (IP); (**B**) pepsin-hydrolysed fraction containing peptides with MW 10–30 kDa (PHp 10–30), (**C**) MW 3–10 kDa (PHp 3–10) and (**D**) MW < 3 kDa (PHp < 3). Data are mean values of six independent experiment ± SD. A statistical analysis was performed using ANOVA followed Dunnett’s post-test (*p* < 0.05).

The cell viability was assayed after 24 h of treatment with the pepsin-hydrolyzed jellyfish peptides PHp 10–30, PHp 3–10 and PHp < 3 and with the undigested fraction IP, at concentrations ranging from 0.05 to 5 µg/mL (Figure 5). No cytotoxic effect and no significant changes of cell viability, as compared to the controls, was evident in keratinocytes treated with all the hydrolyzed protein fractions at all tested concentrations.

**Figure 6:**
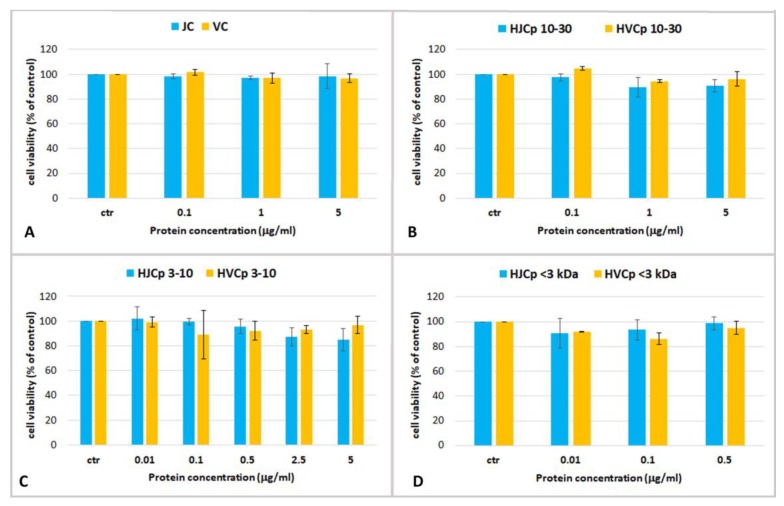
Cell viability of human epidermal keratinocytes (HEKa) treated with different concentrations of (**A**) jellyfish and calf skin collagen (JC and VC); (**B**) collagenase-hydrolysed fraction containing peptides with MW ranging 10–30 kDa (HJCp 10–30 and HVCp 10–30), (**C**) MW 3–10 kDa (HJCp 3–10 and HVCp 3–10) and (**D**) MW < 3 kDa (HJCp < 3 and HVCp < 3). Data are mean values ± SD of six independent experiments. Statistical analysis performed with ANOVA and Dunnett’s test (*p* < 0.05).

The cell viability of HEKa cells was also assayed after a 24 h-treatment with different concentrations of collagenase-hydrolyzed jellyfish peptides: HJCp 10–30, HJCp 3–10 and HJCp < 3 (Figure 6). The effects on HEKa of the bovine collagen fractions (HVCp 10–30, HVCp 3–10 and HVCp < 3), subjected to the identical fractionation procedure, HVCp 10–30, HVCp 3–10 and HVCp < 3, tested at the same concentrations, are also shown. Pepsin-digested fractions before collagenase digestion, JC and VC, were also tested.

Again, no cytotoxic effect and no significant changes of cell viability was observed in keratinocytes treated with all the hydrolyzed protein fractions at all the tested concentrations. In addition, there were no differences between the fractions of jellyfish collagen and the commercial bovine collagen (Figure 6). Over the 24-h course of the treatment, no significant increase in cell proliferation was found in our experiments. The aim of this preliminary work was to establish the maximum non-toxic dose or maximum tolerated dose for jellyfish derived proteins in human keratinocytes, in order to carry out a preliminary screening for bioactive compounds derived from *R. pulmo*.

It is known that bovine collagen increases cell adhesion and proliferation in murine primary keratinocytes [100] and pepsin-solubilized collagen from red sea cucumber (*Stichopus japonicus*) increased the cell migration in wound-healing test, fibronectin synthesis and cell proliferation in human keratinocyte (HaCaT) better that mammalian collagens [101]. A concentration of hydrolyzed fish collagen ranging from 0.76–1.53 µg/mL was found to increase keratinocytes proliferation [102]. To the best of our knowledge, there are no studies about jellyfish collagen and keratinocytes *in vitro*.

In mice, dietary supplementation with *Rhopilema asamushi* jellyfish collagen (JC) and jellyfish collagen hydrolysate showed *in vivo* protective effects on skin photoaging, alleviating the UV-induced changes of antioxidative enzy *in vivo* and *in vitro* mes and the content of glutathione, also protecting skin lipids and hydroxyproline content from the UV radiation damages [37]. Furthermore, collagens enhanced skin immunity, reduced water loss, restored cutaneous collagen and elastin levels and structure, and maintained type III to I collagen ratio in the model of chronic UVA + UVB irradiation of mice [38].

#### 2.3.3. Effect of Fractions of Hydrolysed Jellyfish Collagen on HEKa Cell Cultures Subjected to Oxidative Stress

**Figure 7:**
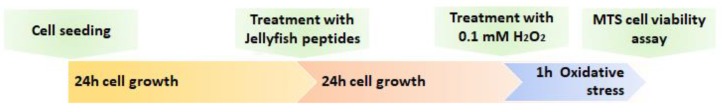
Experimental scheme of the cells treatments with collagen peptides and Hydrogen peroxide (0.1 mM). Controls without treatment and stress induction (1 h with H_2_O_2_) were run for each experiment (*n* = 6). Cells vitality was analyzed using MTS assay, after 1 h of the oxidative stress.

The effect of jellyfish collagen-derived peptides on keratinocytes was also evaluated in co-occurrence of a chemically induced oxidative stress, in order to verify the antioxidant capacity of jellyfish derived peptides in cells. Hydrogen peroxide solution (H_2_O_2_ 0.1 mM) was used to induce reactive oxygen species (ROS) formation in cells. Keratinocytes were pre-treated with the highest concentration of jellyfish peptides for 24 h, and in the last hour of the experiment the H_2_O_2_ solution was added (Figure 7).

**Figure 8:**
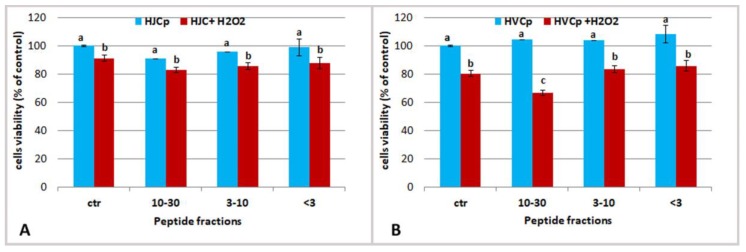
Effect of oxidative stress on HEKa cells of different concentration of jellyfish (**A**) and calf skin (**B**) collagen hydrolyzed peptides MW ranging 30–10 kDa (5 µg/mL), 3–10 kDa (5 µg/mL) and <3 kDa (0.5 µg/mL). Cells viability was valuated 1 h after H_2_O_2_ treatment. Data are mean values ± SD of six independent experiments, analysed with ANOVA and Bonferroni post-test (*p* < 0.05).

Specifically, the fractions HJCp 10–30 and HJCp 3–10 were tested at the protein concentration of 5 µg/mL and the fraction HJCp < 3 was tasted at 0.5 µg/mL (Figure 8). Same concentrations of the peptide fractions derived from bovine collagen (HVCp) were administered in parallel experiments. The main difference between jellyfish and vertebrate collagen peptides was observed when cells were pre-treated with peptides having a MW ranging 10–30 kDa, showing a higher protective effect against oxidative stress due to HJCp 10–30 fraction. Maybe the peptides HJCp 10–30 derived from jellyfish collagen could quench the ROSs molecules, preventing oxidative cell damaging, better than bovine collagen peptides.

Further analysis such as determination of amino acidic composition, biochemical features and health effects will pave the way to better characterize the peptide fractions. Available evidence suggests that protein hydrolysates, including hydrolyzed collagen, from different sources with antioxidant and other functional properties and biological activities, will increasingly receive attention from the large community of researchers working on health food, nutraceuticals and cosmeceuticals industries as well as on processing/preservation technologies.

In conclusion, this work introduces the idea and the methodology for a safe use of proteinaceous compounds from *Rhizostoma pulmo* biomass, by reducing the potential cytotoxic fractions by aqueous extractions and producing antioxidant peptide fractions by protein hydrolyses of the insoluble fraction. A growing scientific evidence base demonstrates that jellyfish can be considered as a valuable source of new bioactive metabolites and the improvements in jellyfish processing technologies will grant the use of abundant jellyfish biomass as a sustainable resource for marine biotechnology applications. We predict that the large natural biomass of *R. pulmo* in the Mediterranean basin will sustain the development of new research and new applications of jellyfish-derived compounds in the cosmeceutical, nutraceutical and pharmaceutical fields.

## 3. Materials and Methods

### 3.1. Chemicals, Materials and Equipment

Amicon^®^ Ultra-15 Centrifugal Filter Devices and acetic acid were purchased from Merck (Darmstadt, Germany). Bovine serum albumin (BSA), phosphate buffered saline (PBS), Pepsin from porcine gastric mucosa (≥2500 U/mg), Collagenase from *Clostridium histolyticum* [0.5–5.0 furylacryloyl-Leu-Gly-Pro-Ala (FALGPA) units/mg solid, ≥125 collagen digestion unit (CDU)/mg solid], Collagen from calf skin (Sigma-Aldrich, Saint Louis, MS, USA), ABTS [2,20-Azinobis (3-ethylben-zothiazoline-6-sulfonic acid) diammonium salt], Cell Freezing Medium with DMSO serum-free, TES(2-{[1,3-Dihydroxy-2-(hydroxymethyl)-2-propanyl]amino}ethanesulfonic acid buffer), Hydrogen peroxide solution and Trypsin-EDTA solution were purchased from Sigma-Aldrich (Milan, Italy). Protein Assay Dye Reagent concentrates, TGX™ FastCast™ Acrylamide Solutions, Protein Standard for Electrophoresis and ChemiDoc™ MP Imaging System, Bio-Rad Protein Assay were purchased from Bio-Rad Laboratories (Munich, Germany). Potassium persulfate (dipotassium peroxdisulfate), 6-hydroxy-2,5,7,8-tetramethylchroman-2-carboxylic acid (Trolox) were purchased from Hoffman-La Roche (Basel, Switzerland). Dulbecco’s phosphate buffered saline (DPBS); Cascade Biologics™ Epilife^®^ with 60 µM calcium; HKGS, were purchased from Life Technologies (Carlsbad, CA, USA). Human Epidermal Keratinocytes adult (HEKa), Trypan blue solution 0.4%, Countess™ automated cell counter and Countess™ cell counting chamber slides were purchased from Invitrogen™ (Carlsbad, CA, USA). MTS CellTiter 96^®^ AQueous Non-Radioactive Cell Proliferation Assay was purchased from Promega (Madison, WI, USA). Infinite M200, quad4 monochromator™ detection system was purchased from Tecan group (Männedorf, Switzerland). Flat and round-bottom 96-well microplates were purchased from Corning (Corning, NY, USA).

### 3.2. Jellyfish Samples

*Rhizostoma pulmo*, Macrì 1778 [67] specimens were collected at Marina di Ginosa (Taranto, Italy) (Supplementary Materials, Video S1), in summers 2017–2018, by means of a nylon landing net with 3.5 cm mesh size, from an open type motorboat, and stored in refrigerated seawater in 100 L barrels for a maximum of 2 h. Specimens were adult both male and female jellyfish with a diameter ranging from 17 to 25 cm. Whole jellyfish were individually frozen in liquid nitrogen and stored at −80 °C. Frozen samples were then lyophilized in a freeze dryer (Freezone 4.5 L Dry System, Labconco Co. Thermo Scientific, Milan, Italy), at −55 °C for 4 days using a chamber pressure of 0.110 mbar and then stored at −20 °C until use. Each lyophilized jellyfish has been made homogeneous (oral arms and umbrella) by mixing its powder, and then the lyophilized powder from 5 individuals was pooled. Six different pools were considered as representative samples and used for independent experiments.

### 3.3. Protein Extraction and Sequential Hydrolysis

**Figure 9:**
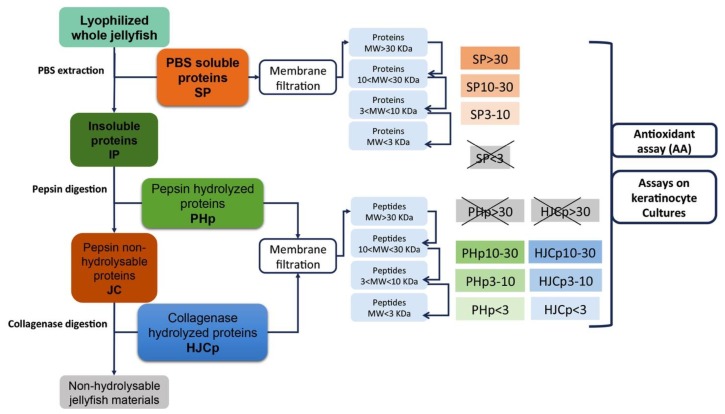
Flow diagram showing the various steps of extraction, hydrolysis and fractionation of the proteins from *Rhizostoma pulmo*.

Lyophilized tissues were ground into a fine powder with liquid nitrogen and 1 g was used as described below (Figure 9). Soluble proteins (SP) were extracted by insoluble material (IP) by gentle stirring of the sample with 16 volumes (*w*/*v*) of PBS, (phosphate buffer saline) pH 7.4, at 4 °C for 2 h and then centrifuged at 9000× *g* for 30 min at 4 °C. Supernatant was separated from the insoluble material (IP) and subjected membrane fractionation as described below. Pellet was subjected to sequential enzymatic hydrolyses by pepsin (1 mg/mL) in 0.5 M acetic acid, using an enzyme/substrate ratio of 1:50 (*w*/*w*) and stirred for 48 h at 4 °C. The digested sample was centrifuged at 9000× *g* for 30 min and the pepsin-hydrolyzed peptides (PHp) were stored for further separations. The pellet was washed two times with bi-distilled water, and subjected to a second digestion with collagenase (6 mg/mL in TES buffer 50 mM, pH 7.4 and 0.36 mM of CaCl_2_) using an enzyme/substrate ratio of 1:50 (*w*/*w*), by stirring 5 h at 37 °C. Collagenase cuts the peptide sequences as –R-Pro-X-Gly-Pro-R where X is generally a neutral amino acid. After hydrolysis, the sample was centrifuged at 9000× *g* for 30 min, and the soluble collagenase-hydrolyzed peptides (HJCp) were stored for further treatments. The pellet of collagenase digestion was considered as not-hydrolysable material. Commercial calf skin collagen (Sigma) was used as control and subjected to the same sequential hydrolysis procedure.

Soluble proteins derived from PBS extraction (SP), pepsin hydrolyzed peptides (PHp) and hydrolyzed collagen peptides (HJCp) were subjected to fractioning by membrane filtration.

### 3.4. Proteins Separation by Membrane Filtration

All the obtained fractions (SP, PHp and HJCp) were separated by membrane filtration in fractions containing peptides with different molecular weight ranges. All the steps were performed at 4 °C. Each sample was filtered using Amicon^®^ Ultra 30K device (Merck) by centrifugation at 4000× *g* to almost total filtration, the retentate contained compounds with MW higher than 30 kDa. The filtrates (containing compounds less than 30 kDa) were further fractionated using Amicon^®^ Ultra 10K device (Merck) by centrifugation at 4000× *g* to obtain the 10–30 kDa fraction in the retentate. Finally, the filtrates containing compounds lower than 10 kDa were centrifuged using Amicon^®^ Ultra 3K device (Merck) at 4000× *g* to obtain in the retentate fractions with 10 < MW < 3 kDa and MW < 3 kDa. Each sample was analyzed for protein content, antioxidant activity and cell culture test.

### 3.5. Protein Content

Total protein content was estimate by Bradford assay [103]. The assay was modified and adapted to round bottom 96-well microplate for Infinite M200, quad4 monochromator™ detection system (Tecan, Männedorf, Switzerland) using bovine serum albumin (BSA) as a standard.

### 3.6. Antioxidant Activity

The antioxidant activity was evaluated by Trolox Equivalent Antioxidant Capacity (TEAC) method adapted for 96-well microplates and Infinite M200 (Tecan, Männedorf, Switzerland), using the radical cation ABTS•+ and Trolox (Hoffman-La Roche) as standard [104,105]. Briefly: 10 µL of each sample was added to 200 µL of ABTS•+ solution, were stirred and the absorbance at 734 nm was read at 6 min [11,106]. Trolox was used as standard and was assayed under the same conditions of the samples. Results were expressed as nmol of Trolox Equivalents per mg of contained proteins (nmol TE/mg protein).

### 3.7. Proteins SDS-PAGE Analysis

Total jellyfish proteins and polypeptides fractions obtained from soluble proteins extracted with PBS were analyzed by SDS-PAGE. A FastCast premixed acrylamide solution 12% was used to prepared gels and “All Blue Precision Plus Protein Standard” (Biorad) was used as molecular weight marker. In order to visualize protein bands, gels were both analyzed by stain-free system with high sensitivity imagined using ChemiDoc™ MP Imaging System (Biorad) and stained with Coomassie Brilliant Blue G-250 (Bio-Rad Protein Assay).

### 3.8. HEKa Cell Culture

Human epidermal keratinocytes, isolated from adult skin (HEKa) were obtained from Cascade BiologicsTM (Gibco^®^) and routinely grown in EpiLife^®^ medium with 60 µM calcium (GIBCO) as described in Leone et al. [10]. Trypan blue dye exclusion and automated counting method by Countess™ was used for routinely cell viability assay and live cell counting. For all experiments, 0.15 × 10^6^ cells/well (75000 cells/mL) were incubated in flat bottom 96-well microplates.

### 3.9. Cell Treatments and Oxidative Stress Induction with H_2_O_2_

All jellyfish protein fractions were diluted in EpiLife^®^ culture medium to reach a final concentration on the cells ranging from 0.05 and 20 µg/mL of proteins/peptides. Soon after dilution, the jellyfish samples were added to cells grown for 24 h in 96-well microplates at 37 °C with 5% CO_2_ (Thermo Forma direct heat CO_2_ incubator). Controls were included in each experiment and in each microplate with medium only (without cells), cells with only medium, and cells with the vehicle (PBS or digestion buffers), at the same final concentration as in the cells treated with the jellyfish samples. For each independent experiment, each treatment, namely each sample, each control and each concentration, was replicated in five technical replicates. Microplates were then incubated for 24 h at 37 °C with 5% CO_2_ (Thermo Forma direct heat CO_2_ incubator).

#### 3.9.1. Cell Treatments with Heat-Denatured Protein

Aliquots of soluble extracted proteins (SP) and the sub-fraction SP > 30 were also heat-denatured by heating at 100 °C for 10 min (Heat Treatment 100 °C) in a water bath, cooled, diluted in EpiLife^®^ culture medium and administrated to the cells.

#### 3.9.2. Cell Treatments with H_2_O_2_

In the experiments for antioxidant activity assay, HEKa cells (0.15 × 10^6^ cells/well) were grown for 24 h to reach 80% of confluence in flat bottom 96-well microplates, and then were treated with the collagen peptides fractions from jellyfish and from bovine collagen at the same concentrations. Two controls with medium and vehicle were also included. After 24 h, 100 µL medium contained H_2_O_2,_ at the final concentration of 0.1 mM were supplied and cells were incubated for 1 h at 37 °C with 5% CO_2_, as reported in Figure 7. Cell viability was assayed by MTS assay soon after the 1 h of treatment.

### 3.10. Cell Viability Assay

MTS Cell viability test was used to establish the effects of the extracted jellyfish compounds. MTS assay was performed using CellTiter 96^®^ AQueous One Solution Reagent (Promega) according to the manufacturer’s instructions. 20 µL of CellTiter 96^®^ AQueous One Solution Reagent were added to each well, the microplates were incubated for 90 min at 37 °C with 5% CO_2_ (Thermo Forma direct heat CO_2_ incubator) and the absorbance was read at 490 nm with Infinite M200. Data were expressed as percentage of the respective controls.

### 4.11. Statistical Analysis

Statistical analyses were performed by Graphpad Prism 6.0. An unpaired Student’s *t*-test was used to compare two groups; analysis of variance (ANOVA) and Dunnett’s *post hoc*-test was applied to compare control with all other treatments, instead a Bonferroni post-test was applied to analyze data in oxidative stress experiments. Differences were considered statistically significant for values *p* < 0.05. All assays were replicated different time (*n* = 6) and data are represented as mean ± standard deviation (SD). The half maximal inhibitory concentration (IC_50_) for fractions was calculated using the same program Graphpad Prism 6.0.

## Figures and Tables

**Table 1:** Protein contents in soluble and insoluble fractions from lyophilized *Rhizostoma pulmo* whole jellyfish samples. Soluble proteins were separated in four sub-fractions at different molecular weight (MW) ranges by membrane filtration. Data expressed as mean ± standard deviation (SD) of six experiments.

Fractions	Protein Concentration	
	mg/g DW ± SD	% Total Proteins
Soluble Proteins (SP)	19.2 ± 1.9	56.3
SP > 30 (MW > 30 kDa)		(52.1)
SP 10–30 (10 kDa < MW < 30 kDa)		(2.3)
SP 3–10 (3 kDa < MW < 10 kDa)		(0.3)
SP < 3 (MW < 3 kDa)		(1.6)
Insoluble Proteins (IP)	14.9 ± 0.9	43.7
Total	34.1 ± 2	100

## Supplementary Materials

The following are available online at https://www.mdpi.com/1660-3397/17/2/134/s1, Video S1: The sampling site of Rhizostoma pulmo jellyfish at Marina di Ginosa, Ionian Sea, Italy (@Antonella Leone CNR-ISPA).
